# Cognitive Performance Measures in Bioelectromagnetic Research - Critical Evaluation and Recommendations

**DOI:** 10.1186/1476-069X-10-10

**Published:** 2011-01-25

**Authors:** Sabine J Regel, Peter Achermann

**Affiliations:** 1 University of Zurich, Institute of Pharmacology & Toxicology, Winterthurerstrasse 190, 8057 Zurich, Switzerland; 2 University of Zurich, Zurich Center for Integrative Human Physiology, Institute of Physiology, Winterthurerstrasse 190, 8057 Zurich, Switzerland; 3 University of Zurich, Neuroscience Center Zurich, Winterthurerstrasse 190, 8057 Zurich, Switzerland

## Abstract

**Background:**

The steady increase of mobile phone usage has led to a rising concern about possible adverse health effects of radio frequency electromagnetic field (RF EMF) exposure at intensities even below the existing safety limits. Accumulating evidence suggests that pulse-modulated RF EMF may alter brain physiology. Yet, whereas effects on the human electroencephalogram in waking and sleep have repeatedly been shown in recent years, results on cognitive performance are inconsistent.

**Methods:**

This review compares 41 provocation studies regarding the effects of RF EMF exposure similar to mobile telephones on cognitive performance measures in humans. The studies were identified via systematic searches of the databases Pub Med and ISI Web of Science and were published in peer-reviewed journals between 1998 and the end of 2009.

**Results:**

Based on a critical discussion within the scope of methodological standards it is concluded that state-of-the-art-methods in bio-electromagnetic research on RF EMF effects and cognition have neither been specified nor fully implemented over the last 10-11 years. The lack of a validated tool, which reliably assesses changes in cognitive performance caused by RF EMF exposure, may contribute to the current inconsistencies in outcomes. The high variety of findings may also be due to methodological issues such as differences in sample size and the composition of study groups, experimental design, exposure setup as well as the exposure conditions, and emphasizes the need for a standardized protocol in bioelectromagnetic research.

**Conclusions:**

At present, no underlying biological mechanism has been identified which mediates the effects on brain functioning as observed in electroencephalographic (EEG) studies. A future aim must be to identify this mechanism as well as a reliable exposure protocol in order to gain more insights into possible behavioral and related health consequences of high-frequency EMF exposure.

## Background

There is a rising concern about possible adverse health effects of radio frequency electromagnetic fields (RF EMF) such as those emitted by mobile telephones. In view of this development, there is an urgent need for investigating the effects of mobile communication systems on brain functioning. Such effects are usually addressed by investigating RF EMF induced changes in the electroencephalogram (EEG) or alterations in cognitive performance. In addition, some imaging studies have been performed to assess possible changes in regional cerebral blood flow (rCBF [1-3]). The best understood effect of RF EMF is to cause heating of the underlying tissue. This heating effect varies with the frequency of the electromagnetic energy as each frequency in the electromagnetic spectrum is absorbed by living tissue at a different rate (specific absorption rate (SAR) in W/kg). To prevent potentially damaging thermal effects international exposure guidelines have been established, limiting the SAR to 2 W/kg for the general public and to 10 W/kg for people undergoing occupational exposure (averaged over 10 g of tissue, [4]). Yet there is increasing evidence supporting the existence of complex non-thermal biological effects of RF EMF at field strengths below exposure limits. Until now, the definite existence and possible extent of these effects is not fully established and the fundamental mechanisms of the interaction between RF EMF exposure and biological matter, in particular the brain, are not understood.

A considerable amount of research has been conducted in recent years concerning short term EMF effects on human brain physiology. So far, most consistent effects were observed with respect to changes in electrical brain activity. Spectral power in the alpha (8-12 Hz) and the sleep spindle frequency range (12-15 Hz) of the EEG are the two most consistently affected variables in waking and non-rapid-eye-movement (non-REM) sleep, respectively (e.g. [2,5-12]; reviewed in [13]). Furthermore, it seems that pulse modulation of the field is crucial for inducing an effect (e.g. [3,11]) which supports the notion of a non-thermal effect.

In contrast to effects on EEG spectral power, effects on cognitive performance measures are rather contradictory. Whereas initially an improvement in terms of decreased reaction times or elevated accuracy scores was reported in response to RF EMF exposure (e.g. [14-16]), more recent studies primarily revealed an impairment of mental abilities (e.g. [17-19]) or no effect at all (e.g. [20-25]). Out of the 41 studies evaluated in this review (Table 1 and 2), employing distinct cognitive tasks of various levels of difficulty, six studies revealed an increase in performance speed [14-16,26-28], and seven studies reported a decrease [11,12,17,29-32]. Likewise, accuracy of performance was reduced [18,19] and elevated [11,12,33,34] in several experiments. Additional findings refer to increased verbal memory and visuospatial working memory capacity [29], a reduction of false alarms [15], a decreased retrieval efficiency [31] and a reduced distance traveled in the Virtual Morris Water Task [35]. More than half of the reviewed studies observed no behavioral changes at all (Table 1 and 2).

**Table 1:** RF EMF effects on cognitive performance in studies with a within-subject design.

Study	RF EMF Exposure			Experimental Design				Cognitive Task(s)				Results
				
	Parameters [Modulation ()]	Side	Duration (min)	Sample	Handedness	Blinding	Cond ≥24 h	**N**^**o**^	Duration (min)	Practice session	When	
				
Aalto et al. 2006* [1]	902 MHz (217 Hz), 577 μs pulse width, 0.25 W mean power, SAR_10 g _= 0.743 W/kg, SAR_peak _= 1.51 W/kg	L	51	12 M	R	DB	C	1	-	-	D	n.s.
Cinel et al. 2008 [64]	a) 888 MHz GSM, SAR_avg _= 1.4 W/kg, SAR_peak _= 11.2 W/kg b) 888 MHz CW, SAR_avg _= 1.4 W/kg											
	Experiment 1:	L+R	~45	44 M, 116 F	-	DB	✓	5	~40	✓	D	n.s.
	Experiment 2:	L+R	~40	52 M, 116 F	-	DB	✓	3	~35	✓	D	↓ RT (Stroop Task, "control" condition)

Croft et al. 2002** [21]	900 MHz (217 Hz), 577 μs pulse width, 3-4 mW mean power (Nokia 5110)	PC	20	16 M, 8 F	R (20) L (4)	SB	C	1	3 (repeated 4 times)	-	D	n.s.

Curcio et al. 2004 [26]	902 MHz (217 Hz), 0.25 W mean power,	L	45	EG1: 5 M, 5 F	R	DB	✓	4	~22	✓	EG1: D	↑ Speed (Acoustic Simple Reaction Time Task)
	SAR = 0.5 W/kg (Moto Timeport 260)			EG2: 5 M, 5 F							EG2: A	↑ Speed to targets (Acoustic Choice Reaction Time Task)

Curcio et al. 2008 [20]	902.40 MHz (217 Hz), 0.25 W mean power, SAR = 0.5 W/kg (Moto Timeport 260)	R	15 (repeated 3 times)	12 M, 12 F	R	DB	✓	2	10 (repeated 3 times)	✓	D	n.s.

Eliyahu et al. 2006 [30]	890.2 MHz, 577 μs pulse width, 2 W peak power (Nokia 5110)	L+R	~120	36 M	R	SB	-	4	~120	✓	D	↑ RT with left hand (left side exposure, 2^nd ^ session, Spatial Item Recognition Task)

Freude et al. 2000** [49]	916.2 MHz (217 Hz), 577 μs pulse width, 2.8 W peak power, SAR_1 g _= 1.42 W/kg, SAR_10 g _= 0.882 W/kg											
	Experiment 1:	L	-	20 M	R	SB	C	1	-	✓	D	n.s.
	Experiment 2:	L	-	19 M	R	SB	C	3	-	✓	D	n.s.

Haarala et al. 2003a* [60]	902 MHz (217 Hz), 577 μs pulse width, 0.25 W mean power, SAR_10 g _= 0.993 W/kg, SAR_peak _= 2 W/kg (Moto Timeport 260)	L	~45	14 M	R	DB	C	4	-	-	D	n.s.

Haarala et al. 2003b [23]	902 MHz (217 Hz), 577 μs pulse width, 0.25 W mean power, SAR_1 g _= 0.88 W/kg, SAR_peak _= 1.2 W/kg											
	Experiment 1:	L	~65	16 M, 16 F	R	DB	✓	9	~65	✓	D	n.s.
	Experiment 2:	L	~65	16 M, 16 F	R	DB	✓	9	~65	✓	D	n.s.

Haarala et al. 2004 [24]	902 MHz (217 Hz), 577 μs pulse width, 0.25 W mean power, SAR_10 g _= 0.99 W/kg, SAR_peak _= 2.07 W/kg (Nokia 6110)											
	Experiment 1:	L	~65	16 M, 16 F	R	DB	✓	4	~65	✓	D	n.s.
	Experiment 2:	L	~65	16 M, 16 F	R	DB	✓	4	~65	✓	D	n.s.

Haarala et al. 2005 [22]	902 MHz (217 Hz), 577 μs pulse width, 0.25 W mean power, SAR_1 g _= 1.44 W/kg, SAR_10 g _= 0.99 W/kg, SAR_peak _= 2.07 W/kg	L	~50	16 B, 16 G	R	DB	✓	8	~50	✓	D	n.s.

Haarala et al. 2007 [48]	a) 902 MHz (217 Hz), 577 μs pulse width, 0.25 W mean power, b) 902 MHz CW, 0.25 W power	L+R	~90	48 M	R	DB	✓	9	~45	✓	D	n.s.
	a) b) SAR_1 g _= 1.1 W/kg SAR_10 g _= 0.738 W/kg, SAR_peak _= 1.18 W/kg (Nokia 6110)											

Hamblin et al. 2004** [17]	894.6 MHz (217 Hz), 576 μs pulse width, 2 W peak power, SAR = 0.87 W/kg (estimated, modified Nokia 6110)	R	60	4 M, 8 F	R	SB	✓	3	~30	✓	D	↑ RT (Auditory Oddball task)

Hamblin et al. 2006** [25]	895 MHz (217 Hz), 576 μs pulse width, 2 W peak power, SAR_10 g _= 0.11 W/kg (modified Nokia 6110)	R+L	30	46 M, 74 F	R (108) L (12)	DB	✓	3	15	✓	D	n.s. (one result not reported)

Hinrichs & Heinze 2004** [58]	1870 MHz (217 Hz), 0.125 W mean power, 1 W peak power SAR_1 g _= 1.14 W/kg, SAR_10 g _= 0.61 W/kg	L	30	2 M, 10 F	-	DB	✓	1	10 (at end of exposure)	-	D	n.s.

Jech et al. 2001** [27]	900 MHz (2, 8, 217 Hz), 577 μs pulse width, 2 W max. power, SAR_10 g _= 0.06 W/kg, (Moto d520)	R	45	9 M, 13 F	R (20) L (1) A (1)	DB	✓	1	12.5	-	D	↓ RT to targets

Keetley et al. 2006 [31]	0.25 W mean power (Nokia p6110) Sham: standby-mode	L	60	58 M, 62 F	-	DB	✓	8	~30	-	B, D	↓ Performance variable 7, ↓ retrieval efficiency (Rey's Audio Visual Learning Test)
												↓ Performance (TMT A), ↑ performance (TMT B) ↑ RT (RT Task) ↑ RT (Choice RT Task)

Kleinlogel et al. 2008** [63]	a) 900 MHz (GSM, 2, 8, 217 Hz), b) 1950 MHz (UMTS), GSM and UMTS high: psSAR_10 g _= 1 W/kg UMTS low: psSAR_10 g _= 0.1 W/kg	L	30	15 M	R	DB	✓	1	11	✓	D	n.s.

Koivisto et al. 2000a [14]	902 MHz (217 Hz), 577 μs pulse width, 0.25 W mean power	L	30	24 M, 24 F	R	SB	C	4	~30	✓	D	↓ RT to targets (3-Back Task)

Koivisto et al. 2000b [15]	902 MHz (217 Hz), 577 μs pulse width, 0.25 W mean power	L	60	24 M, 24 F	R	SB	✓	12	~60	✓	D	↓ RT (Simple RT Task) ↓ RT, ↓ false alarms (Vigilance Task) ↓ Subtraction time (Subtraction Task)

Krause et al. 2000a** [51]	902 MHz (217 Hz), 577 μs pulse width, 0.25 W mean power (Nokia phone)	R	30	8 M, 8 F	R	SB	C	1	~30	-	D	n.s.

Krause et al. 2000b** [54]	902 MHz (217 Hz), 577 μs pulse width, 0.25 W mean power, SAR < 2 W/kg (manufacturer info) (Nokia phone)	R	30	12 M, 12 F	R	SB	C	3	~30	-	D	n.s.

Krause et al. 2004** [18]	902 MHz (217 Hz), 577 μs pulse width, 0.25 W mean power, SAR_1 g _= 0.878 mW/kg, SAR_10 g _= 0.648 mW/kg (Nokia phone)	L	30	12 M, 12 F	R	DB	C	1	~30	-	D	↑ Error rates (mean percentage of incorrect answers; Modified Sternberg Task)

Krause et al. 2007** [50]	a) 902 MHz (217 Hz) 577 μs pulse width, 0.25 W mean power b) 902 MHz CW, 0.25 W mean power a) b) SAR_1 g _= 1.1 W/kg, SAR_10 g _= 0.738 W/kg, SAR_peak _= 1.18 W/kg (Nokia 6110)											
	Experiment 1:	L+R	2 × ~27	36 M	R	DB	✓	1	~27	-	D	n.s.
	Experiment 2:	L+R	2 × ~40	36 M	R	DB	✓	4	~40	-	D	n.s.

Preece et al. 1999 [16]	a) 915 MHz (217 Hz), 0.25 W mean power, b) 915 MHz analogue, 1 W mean power											
	Experiment 1:	L	~25-30	9 M, 9 F	R (14) L (4)	DB	✓	10	-	✓	D	↓ RT (Choice RT Task) (exposure b))
	Experiment 2:	L	~25-30	9 M, 9 F	R (16) L (3)	DB	✓	10	-	✓	D	↓ RT (Choice RT Task) (exposure b))

Preece et al. 2005 [52]	902 MHz, a) 0 W power b) 0.25 W peak power c) 2 W peak power SAR_max.brain _= 0.28 W/kg (Nokia 3110)	L	30	9 B, 9 G	-	DB	✓	16	30-35	✓	D	n.s. (after Bonferroni correction)

Regel et al. 2007a [11]	a) 900 MHz (2, 8, 217, 1736 Hz), 12.5% duty cycle b) 900 MHz CW a) b) psSAR_10 g _= 1 W/kg	L	30	24 M	R	DB	✓	5	15 (repeated twice)	✓	D	↓ Speed (2-, 3-Back Task) ↑ Accuracy (3-Back Task) (exposure a))

Regel et al. 2007b [12]	900 MHz (2, 8, 217, 1736 Hz), 12.5% duty cycle a) psSAR_10 g _= 0.2 W/kg b) psSAR_10 g _= 5 W/kg	L	30	15 M	R	DB	✓	5	15 (repeated twice)	✓	D	↓ Speed (1-Back Task, dose-dependent) ↑ Accuracy (1^st ^session 2-Back at 0.2 W/kg)

Rodina et al. 2005 [19]	450 MHz (7 Hz), 50% duty cycle, 1 W output power, SAR = 0.0095 W/kg	R	8 × 5-7	4 M, 6 F	L+R	SB	C	1	3-5 (repeated 8 times)	✓	D	↑ Errors (Visual Masking Task)

Russo et al. 2006 [53]	a) 888 MHz GSM, SAR_avg _= 1.4 W/kg, SAR_peak _= 11.2 W/kg b) 888 MHz CW, SAR_avg _= 1.4 W/kg Sham SAR < 0.002 W/kg	a) b) R+L	35-40	a) b) 84 69 M, 99 F	-	DB	✓	4	~35-40	✓	D	n.s.

Schmid et al. 2005 [59]	1970 MHz (5 MHz), a) High exp: avSAR_1 g _= 0.63 W/kg avSAR_10 g _= 0.37 W/kg b) Low exp: 1/10 of High exp c) Sham ≥50 dB below Low exp	L	-	29 M, 29F	-	DB	C	4	-	✓	D	n.s.

Terao et al. 2006 [57]	800 MHz (50 Hz), 6.7 ms pulse width, 270 mW mean power, SAR_10 g _= 0.054 ± 0.02 W/kg	R	30	9 M, 7 F	R	DB	✓	1	6-7	✓	B, A	n.s.

Unterlechner et al. 2007 [61]	1970 MHz (5 MHz), a) High exp: avSAR_1 g _= 0.63 W/kg avSAR_10 g _= 0.37 W/kg b) Low exp: 1/10 of High exp c) Sham ≥50 dB below Low exp	L	-	20 M, 20F	R (87.5%) L/A (12,5%)	DB	C	4	~30 (repeated 3 times)	✓	D	n.s.

Study: * Experiments on regional cerebral blood flow (rCBF); ** Experiments on event related potentials (ERP)

RF EMF Exposure: (av/ps) SAR: (average/peak spatial) specific absorption rate; L: left side exposure; R: right side exposure; L+R: left and right side exposure; PC: exposure over posterior cortex; GSM: Global System for Mobile Communications; UMTS: Universal Mobile Telecommunications System; CW: continuous wave (carrier frequency only)

Experimental Design: M: males; F: females; B: boys; G: girls; Handedness: R: right handed, L: left handed, A: ambidextrous; SB: single blind; DB: double blind; Cond: exposure conditions applied ≥24 h apart; C: exposure conditions applied consecutively; EG: experimental group

Cognitive Tasks: N^o^: number of tasks applied; B: task applied before exposure; D: task applied during exposure; A: task applied after exposure; RT: reaction times

Results: ↑: significant increase; ↓: significant decrease; n.s.: no significant effect (compared to sham)

✓: accomplished; - not accomplished/specified

**Table 2:** RF EMF effects on cognitive performance in studies with a between-group design.

Study	RF EMF Exposure			Experimental Design					Cognitive Task(s)				Results
				
	Parameters [Modulation ()]	Side	Duration (min)	Sample			Handedness	Blinding	N^o^	Duration (min)	Practice	Time point	
				
Bessett et al. 2005 [47]	900 MHz (217 Hz), 576 μs pulse width, SAR = 0.54 W/kg	L+R	120/day (5days per week, 4 weeks)	EG: 14 M, 14 F		CG: 13 M, 14 F	R (47) L (8)	DB	22	~120	-	B, D, A	n.s.
Edelstyn & Oldershaw 2002 [29]	900 MHz, SAR = 1.19 W/kg	L	30	EG: 19		CG: 19	R	SB	6	~8	-	B, A	After 15 min exposure:
													↑ Verbal memory capacity (Digit Span Forwards)
													↓ Visuospatial working memory capacity (Spatial Span Backwards)
													↑ Sustained attention/↓ processing speed (Serial Subtraction)

Fritzer et al. 2007 [43]	900 MHz (2, 8, 217, 1736 Hz), SAR = 24 mW/kg (whole body)	T	405- 525/night (6 nights)	EG: 10 M		CG: 10 M	-	-	7	-	-	B, A	n.s.

Lass et al. 2002 [33]	450 MHz (7 Hz), 50% duty cycle, 1 W output power SAR = 0.0095 W/kg	R	10-20	EG: 50		CG: 50	-	SB	3	10-20	-	D	↑ Variances of errors (Modified Trail Making Test B)
													↓ Errors (Visual Short Term Memory Task)
													↑ Variances of errors (Symbol Digit Modality Test)

Lee et al. 2003 [28]	1900 MHz (Nokia 3210)	R	30	EG: 39		CG: 39	R	SB	3	~25 (repeated twice)	-	D	↓ RT (Sustained Attention To Response Task)

Luria et al. 2009 [32]	890.2 MHz, 577 μs pulse width, 2 W peak power (Nokia 5110)	L+R	~60	EG1 16 M	EG2 16 M	CG 16 M	R	SB	1	~5 (repeated 12 times)	✓	D	↑ RT with right hand (left side exposure, 1^st ^two blocks, Spatial Item Recognition Task)

Smythe & Costall 2003 [34]	1800 MHz, SAR = 0.79 W/kg (Ericsson A2618s)	L	15	EG1 8 M 9 F	EG2 15 M 11 F	EG3 10 M 9 F	R	SB	1	3	-	D	↑ Spatial accuracy in males (Spatial Word Recall Task)

Wiholm et al. 2009 [35]	884 MHz (GSM), psSAR_10 g _= 1.4 W/kg (time-averaged)	L	150	EG1: 23 9 M 14 F		EG2: 19 12 M 7 F	-	DB	1	-	-	B, A	↓ Distance traveled in Virtual Morris Water Task (EG1)

RF EMF Exposure: (ps) SAR: (peak spatial) specific absorption rate; L: left side exposure; R: right side exposure; L+R: left and right side exposure; T: exposure of top of head; GSM: Global System for Mobile Communications

Experimental Design: EG: Experimental group; CG: Control group; M: males; F: females; Handedness: R: right handed, L: left handed; SB: single blind; DB: double blind

Cognitive Tasks: N^o^: number of tasks applied; B: task applied before exposure; D: task applied during exposure; A: task applied after exposure; RT: reaction times

Results: ↑: significant increase; ↓: significant decrease; n.s.: no significant effect (compared to sham)

✓: accomplished; - not accomplished/specified

Results of a recent meta-analysis suggested that RF EMF may have a small impact on human attention and working memory [36]. In contrast, Valentini and colleagues [37], using a similar approach, reported that mobile phone-like EMF do not seem to induce relevant cognitive and psychomotor effects. One major aspect complicating the comparisons of studies and potentially leading to such contradictory results is the wide variety of methodologies used to assess cognitive performance, such as a multitude of measuring tools and varying exposure conditions, which significantly hamper the interpretation of the results and comparisons among studies. As a result, and despite the growing number of studies, the findings become more and more inconsistent.

The present review critically evaluates the contradictory and partly non-reproducible cognitive outcomes reported in human laboratory studies, which are attributed to exposure to mobile phone communication technology. Whereas a recent publication mainly focused on a detailed summary of the current scientific literature and results [38], here we mainly concentrate on methodological considerations (cognitive tasks as a measuring instrument, materials and methods such as experimental design and sample size, exposure conditions and associated dosimetry) and discuss the necessity of minimal requirements in bioelectromagnetic research, which are often not met in a satisfactory way. An overview of the effects of RF EMF exposure on cognitive performance was summarized above. Details on all publications which have been included in this review are listed in Tables 1 and 2. In the following, the literature is discussed from a methodological viewpoint, subdivided into several chapters, with the most important points summarized in the conclusions. In addition, relevant issues, their impact and importance as well as recommendations how to address them are listed in Table 3.

**Table 3:** List of issues that need consideration when designing bioelectromagnetic studies on cognitive performance measures - Impact, importance and recommendations.

Issue	Impact	Importance	Recommendation(s)
Task type	Detection of effect	High	Use tasks which previously revealed effects and/or address the same cognitive variable (i.e. 'selective attention', 'working memory'); select task related to exposed brain region

Input and response modalities	Comparability between studies	Medium	Use modalities applied in tasks that previously revealed effects

Learning effects	Error variance and type II error	Medium	Include practice sessions; apply conditions in a crossover design; use parallel test forms or random or pseudo-random sequences

Task specificity	Detection of effect	High	Select task related to exposed brain region; if appropriate correct for multiple comparisons^a^

Timing of tasks, task order and task duration	Development of effect; comparability between studies; alertness and motivation	High	Apply tasks with varying difficulty repeatedly in the same order; chose task difficulty and duration wisely to obviate fatigue and/or motivational loss

Study population and sample size	Effect size and significance; external validity	High	Run power analysis to get an indication of sufficiently large sample size; use homogeneous group for small sample sizes; apply proper matching procedures for two or more study groups; characterize study population

Handedness	Comparability between studies; homogeneity of study group(s)	Low to Medium^b^	Include either right or left handers

Inclusion criteria and exclusion criteria	Detection of effect; comparability between studies; homogeneity of study group(s)	Medium to High	Define clear criteria prior and within a study according to the subject of investigation

Confounding factors	Error variance and type II error	High	Control for confounding factors as much as possible experimentally; apply randomization; check compliance of participants to predefined requirements

Experimental design and blinding	Detection of effect	High	Use within-subject, cross-over design if possible; double blinding is mandatory

Exposure conditions	Detection of effect; effect size and significance; interpretation	High	Ensure standardized reproducible exposure conditions; document setup and technical specifications (including signal characteristics); determine exposed brain areas

Field conditions and dosimetry	Interpretation	High	Use field intensities close to exposure limits; provide clear definition of field conditions; dosimetry and sham condition (no field) mandatory

Exposure duration and carryover effects	Interpretation; detection of effect	High	Allow for sufficient exposure duration; consider potential carryover effects in a crossover design; allow for sufficient time interval ('washout period') between conditions

It must be noted that Table 3 contains generalized recommendations only and reflects the main issues which should be considered when designing a new study investigating RF EMF effects on mental processing; however, due to the complexity of each issue the recommendations may not fit all purposes by default. For a more elaborate discussion of each issue and the corresponding implications the reader is referred to the respective chapter of the review.

^a ^A substantial sample size is needed to adequately perform such a correction; ^b ^of high importance when assessing motor reaction times with a cognitive task.

## Literature selection

Literature was collected by extensive online-searches of Pub Med and Web of Science databases using combinations of the following terms: "electromagnetic field", "mobile phone", "cellular phone", "microwave radiation" "EMF exposure", "cognitive performance", "cognitive function", "memory", "attention". Only human provocation studies (controlled experimental exposure studies) including high frequency, handset-like GSM (Global System for Mobile Communication) or UMTS (Universal Mobile Telecommunication System) exposure published in peer-reviewed journals were considered. As a minimum quality requirement, studies had to indicate whether a single- or double-blind design was applied, describe the basic exposure conditions and include a sham control condition ("no field") for the purpose of comparison.

Altogether 48 studies dealing with RF EMF effects on cognitive performance were identified, published between 1998 and the end of 2009. Thereof, 26 studies specifically focused on performance measures (e.g. reaction times, accuracy of performance) in various cognitive tasks. Fourteen studies were run as combined experiments on electrophysiology and cognition, in which human brain oscillatory activity was assessed during cognitive processing (event related potentials, ERP). Four of these did not report on the cognitive outcomes and were not included [39-42]. One pilot study and two provocation studies assessed the effects on sleep and waking and neuropsychological/cognitive variables [11,12,43]. Finally, two studies recorded cognitive performance while measuring RF EMF induced changes on rCBF. Three studies did not meet the mentioned minimal scientific requirements (i.e., acceptable specification of exposure, blinding and test conditions, [44-46]) and therefore were not included.

## Cognitive tasks

### Task type, input and response modalities

Efficient information processing is of great importance in daily life and cognitive tasks of different complexity can reveal intellectual disturbances in response to experimental manipulations. RF EMF research focused almost entirely on behavioral data, such as response times or error rates, to draw inferences about cognitive constructs such as "attention" and "working memory". It is important to stress that most research groups did not indicate why they applied a specific cognitive task. Some researchers made use of already existing test batteries (e.g. [15,22,23,47,48]), others adopted cognitive paradigms of related disciplines (e.g. [19,33]) or based their choice upon previously reported effects in RF EMF research (e.g. [11,12]). Up to date simple and complex reaction time tasks, tasks on selective or divided attention or attention capacity, vigilance and (working) memory have been applied (see Table 1 and 2). Behavioral performance in ERP-studies has mainly been assessed by visual or auditory oddball, memory or discrimination tasks (e.g. [17,18,21,25,27,49-51]).

Unfortunately, cognitive performance tests which reliably measure effects of RF EMF exposure (if existing) are still lacking. Future experiments may preferably follow up on cognitive tasks which previously revealed an effect as this may help to identify a common variable or type of task especially sensitive to RF EMF. The sensory modality of the stimuli (i.e., any of the various types of sensations, such as vision or hearing) used in a cognitive task often varies within and/or between tasks and across studies. However, on closer inspection it appears that significant effects occur unpredictably and independent of task type or input modality. The type of response which is required to react to a stimulus also widely differs between experiments. Mainly computerized tasks were applied (e.g. [14,15,24,33]), with simple motor responses (e.g. pressing a button with one finger) being the most often recorded variable (e.g. [14,15,52]). A high variability exists, however, with respect to the hand and/or the fingers used to respond to a specific stimulus (e.g. [14-16,31,32]). Responses were recorded by pressing corresponding buttons on a keyboard (e.g. [23,24,30,48,53]), on a response or keypad [23,48,54], or on a specific response box (e.g. [11,12,16]) as well as by using a two-button mouse [50]. Some researchers used paper and pencil versions of cognitive tasks [28], and in addition to motor responses, also verbal answers have been assessed (e.g. [24,53]). Assessing different kinds of responses within the same type of task but across different experiments complicates the interpretation of the results. From a methodological point of view it makes sense to hold on to the same response modality used previously (i.e. if the task has been used before) to somehow control for a potential influence of the response modality on the data.

### Learning effects and task specificity

The concept of a "learning curve" was introduced by the 19th-century German psychologist Hermann Ebbinghaus in his study of the efficiency of memorization. Originally, it assumes an asymptotic-like behavior referring to a quick progress in learning during the initial stages of the practical application of a test, followed by gradually smaller improvements with further practice. A better task performance over time is especially important to consider in studies lacking a fully balanced experimental design. However, it must be noted that even a fully counterbalanced design does not account for the problem that the learning effect increases the error variance and leads to more type II error. Practice sessions prior to the beginning of an experiment are advisable in order to minimize the bias of measuring a learning effect rather than a treatment effect, yet their implementation is only mentioned in about 50% of the reviewed papers (see Table 1 and 2). Also, only three groups made use of parallel test forms in their repeated measures in order to obviate learning effects due to recurrent performance of exactly the same task in the course of an experiment [16,25,31]. A different approach has been used for one specific and frequently used working memory test procedure, the N-back task, for which most research groups applied visual stimuli lists which included random or pseudorandom sequences of letters (e.g. [11,12,24,50,54]).

To date, only single tasks, but not a whole test battery yielded significant results [15,16,22,23,47,52]. Most importantly, the reported effects do not seem to depend on a specific type of task (e.g. attention, memory), the sensory modality it is addressing (visual, auditory or verbal material) or its general complexity (e.g. simple reaction time task, N-back task). Such non-specificity, for example an increase in processing speed in one task and a decrease in another task, is difficult to interpret. As discussed in Regel et al. [55], it is most likely that either the tasks are not sensitive enough to consistently reveal an induced effect or that significant performance changes might be chance effects as most authors did not adjust their p-values for multiple testing. This assumption gains further support by the lack of reproducibility of previous results with improved methodology, as seen in several follow-up studies (e.g. [18,23,24,52]). However, it must be noted that though correction for multiple comparisons can be used to reduce the likelihood of false positive results, a substantial sample size is needed to adequately perform such a correction.

### Timing of tasks, task order and task duration

The timing of a task plays an important role in bioelectromagnetic research. Possible effects of RF EMF exposure may be immediate but also delayed in time (for a review see [56]). It cannot be excluded that some of the negative results that emerged may have resulted from the fact that a task was actually applied before or after a measurable effect had developed. It is very difficult to find the right balance between exposure and the critical point in time to assess cognitive functioning (i.e., beginning and ending of a certain task). In the reviewed literature, the majority of studies assessed performance during exposure (see Table 1 and 2), yet it seems that in a few cases the task duration did not necessarily match or even exceeded the exposure duration (e.g. [22-24]). Performance was also measured before and/or after exposure [29,35,43,57], before and during the second part of exposure [31], before, during and after exposure [47], during the last 10 minutes of exposure [11,12,58], as well as exclusively after exposure [26]. Hence, it cannot be excluded that the current inconsistencies in cognitive outcomes may at least be partially due to the high variability in timing of tasks with respect to exposure. In order to detect additional effects possibly outlasting the exposure period, it may be advisable to repeatedly apply the respective cognitive tasks and continuously record performance measures throughout as well as for some time after exposure. The task order itself constitutes an additional important interfering variable. Fifteen research groups included a single task in their study (see Table 1 and 2); four of them applied it repeatedly during the experimental sessions (2, 4, 8 and 12 times [19,21,32,50]). All other experiments included two or more tasks in their experimental design (see Table 1 and 2). From these, nine studies applied the tasks once [16,26,28,33,48,52,59] or twice [11,12] during one exposure session, respectively, always in a fixed order. In three studies a fixed task order appears likely, but is not explicitly stated [43,47,49]. Nine groups made use of a counterbalanced (Latin square) design by randomizing task order and exposure condition across participants [15,22,23,25,29-31,48,53]. Yet, if indeed a certain exposure duration is needed to induce an observable effect (e.g. [11]), tasks which are applied at the very beginning of an exposure may not show a performance change merely due to their early timing within the experimental procedure. Vice versa, tasks which are introduced at the very end of an exposure, might well be properly timed, however, simply not be appropriate to measure an effect (e.g. an effect on the auditory system will not be picked up if a visual task is used). Moreover, an effect may be missed due to the fact that it is only measurable within a certain "time window" during exposure. Therefore, a counterbalanced design, though methodologically reasonable, even increases the likelihood that a significant effect will be randomly distributed among tasks, leading to a type II error.

The duration of a cognitive task or test battery might influence motivation, alertness or vigilance and therefore performance parameters. This is especially important to consider when aiming at information on processing speed. Task duration ranged from three minutes [49] to approximately two hours [47]. Indications exist that a certain exposure duration might be necessary to provoke an effect [11]. Accordingly, it is questionable whether short tasks of a few minutes are capable to reveal induced performance changes at all. Processing of long-lasting tasks might on the other hand promote monotony and thereby influence performance levels. Especially very simple or very difficult tasks might induce motivational loss and/or fatigue in subjects and thus accelerate potential alterations. For example, a simple reaction time task which requires nothing but a fast motor response will presumably attract attention for a rather short period of time before boredom sets in and the participant gets distracted. Importantly, this may result in altered performance levels unrelated to the actual exposure condition. Complex tasks on the other hand might ensure a participant's sustained attention, however, high concentration levels over a long period of time may provoke fatigue and potentially lead to an increase in error rates. Creating diversity by applying several different tasks with a varying degree of difficulty and appropriate task durations may help to ensure the participant's sustained attention during the experimental session.

## Materials and methods

### Study population and sample size

In any experiment we aim at drawing conclusions and making generalizations about a population. As the population is too large to study in its entirety, a sample with a wide spectrum of sociodemographic characteristics that is meant to be representative of the population is examined. Yet, it may be possible that subgroups of subjects rather than a whole population react especially sensitive to a certain treatment. In this respect, it is speculated whether RF EMF radiation poses a higher risk to children, who are in an early stage of central nervous system (CNS) maturation. This remains hypothetical until more data has been collected.

The determination of an appropriate sample size for an experiment is important and should be in accordance with a reasonable effect size. This requires that the number of participants must be sufficiently large to be able to detect potential effects; at the same time it must not be too large to prevent detection of very small effects.

A very heterogeneous group of subjects may mask an existing effect and in most instances major differences between study populations may be a reason for the inconsistencies in the cognitive outcomes reported. Though heterogeneity is not likely to be a problem in large study populations, with small samples it should be preferentially aimed at a highly homogeneous group. In any case, an adequate description of the study population is mandatory. In a between-group design ideally proper matching procedures are applied to ensure that groups are similar with regard to any factors that might distort or confound a relationship that is being studied. The studies performed to date mainly assessed the responses to RF EMF exposure in young and middle-aged male and female subjects. The age range of the participants fluctuates considerably within and between studies (~ 17-70 years), the average age of a study sample rarely exceeded 30 years [16,31]. Though in all but one study [27] healthy participants were investigated, some studies recruited a specific sub-sample (e.g. young healthy male students, children) for their experiments (e.g. [11,12,48,52]) while others aimed to test a broader sample of the general population (e.g. both genders of a large age cohort, [16,31]). Interestingly, so far no study included exclusively female participants. In contrast, 11 experiments included males only (see Table 1 and 2) aged on average between 22.1 [11] and 28.5 years [43]. Both genders were tested in altogether 27 studies, the age span ranging from 10.2 [52] to 70 years [31]. In three studies, the gender [29,33] and the proportional distribution of men and women in the two investigated groups [28] were not further specified. Also, in many cases males and females were not equally distributed within the study sample (see Table 1 and 2). As it cannot be excluded that RF EMF exposure acts differently on males and females, in particular of different ages, present results might have been influenced by the unbalanced gender/age composition of the study groups.

In cognitive research, assessing motor reaction times often constitutes the variable of choice to gain information on mental ability changes due to a treatment. Accordingly, the handedness of the subjects is an important factor which has to be controlled for in an experimental setting. It may be advisable to only recruit left- or right-handers from the outset to exclude a possible bias. The majority of experiments so far involved right-handed participants (see Table 1 and 2). Left-handers and/or ambidextrous subjects have been included in seven studies, however one paper did not further specify the distribution of right and left-handers [19,53]. Nine research groups did not state the subject's handedness at all (see Table 1 and 2). In general inhomogeneous groups of subjects should be carefully inspected with respect to their most striking differences. This can be achieved by either considering these factors in the statistical model or by testing the different groups against each other before finally pooling the data.

Apart from the specific characteristics of a study population a power analysis to estimate an adequate sample size is recommended. In bioelectromagnetic research the effect size is presumably small with high interindividual variability and several key values need to be considered (e.g. expected effect size of the treatment, variability of the outcome measure in the populations, type I and type II error rates). So far few studies included large numbers of subjects in their experiment [25,31,53] and/or performed a power analysis prior to its initiation [25,53]. In a within-subject (crossover) design (Table 1) including male and/or female subjects, the sample size ranged from 10 [19] to 168 subjects [53]. When two different groups were involved (Table 2), at the minimum 10 subjects [26,43] and at the maximum 50 subjects [33] were included, respectively. A power analysis prior to an experiment increases the probability to detect an existing effect. However, as the variances are unknown or at best extrapolated from previous experiments, a power analysis is probabilistic itself and not a guarantor to prevent a type II error. Though no formal standards exist, power is generally expected to take a value of at least 0.80 in order to detect a reasonable difference from the null hypothesis. Ideally, both, alpha and beta error rates, should be kept low in this procedure. Power analyses might in particular be helpful in case of null effects as they may provide an indication whether an effect possibly remained undetected due to an undervalued sample size.

### Inclusion criteria, exclusion criteria and confounding factors

Experimental research aims to establish a causal relationship between two variables. In this regard, a continuous problem is the risk that a third variable is actually causing the observed effect. Defining appropriate inclusion and exclusion criteria prior to an experiment as well as establishing certain criteria within the study procedure may reduce this risk considerably. Yet, it is very difficult to grade existing publications according to the mentioned criteria. Scanning the literature, it is often difficult to distinguish inclusion/exclusion criteria from the "mere" description of the study population. Some research groups considered distinctive inclusion/exclusion criteria according to the object of investigation. For example, because they used auditory tasks in their experiments, several scientists presupposed normal hearing of their participants [17,18,21,34,47,51,54]. For the same reason, others, using visual stimuli in their tests, attached importance to normal or corrected-to-normal vision in their study sample [19,25,34,49,53,58,59]. Also, some studies included native speakers only [15,18,22-24,28,48,60], presumably to obviate possible language barriers.

Interestingly, only few research groups commented on the general calling habits of their participants. Yet, in this special context of research, the amount of mobile phone usage constitutes a very important variable which may contribute to the lack in homogeneity within a study sample. The subjects in the studies of Regel et al. [11,12] were selected on the basis of using the mobile telephone less than one hour per week. No calls were allowed until an experimental block was completed. Subjects of Besset et al. [47] were reported to usually use their phones less than 10 minutes per day. Fritzer et al. [43] mention "little use of mobile telephones" in their publication without providing further details.

Besides any fixed criteria prior to a study involvement, sometimes also special requirements within the experiment itself are demanded. For instance, no caffeine and/or alcohol was allowed for three days before the experiment [11,12], during the preceding 24 hours before the testing session [29,48], on the day of the experiment [19] or in the 10 hours prior to the recording [26]. While thus creating a more controlled environment, some restrictions will increase type II variance. This may be the case for example if subjects are not carefully preselected in terms of their caffeine consumption prior to an experiment (i.e. only low or moderate caffeine consumers are included). Indeed a heavy coffee drinker who has to abstain from caffeine during an experiment may experience withdrawal effects during testing which will affect performance levels.

Sleep history and sleep-wake behavior prior to the study has been controlled for in altogether eight studies. Participants did not report any sleep complaints [12,26] or sleep less than usual [29], had adequate sleep in the night prior to the experiment [30,32], maintained a regular sleep-wake-cycle [43] for three days before the study enrollment [11,12] with no shift-work and no presence or history of sleep disorders [43,61], chronic sleep deprivation [61] or sleep apnea, nocturnal myoclonus or low sleep efficiency [12].

All experiments should be performed under highly standardized conditions, including basic principles such as constant ambient light and temperature conditions, identical test procedures in case of repeated assessment or controlling for possible time of day (circadian) influences. A major problem is that confounding variables are not always known or measurable. In this case, the best solution may be randomization as it increases the probability that all confounding variables, known and unknown will be equally distributed across a study group. Successful randomization usually requires a sufficiently large number of subjects, though. Confounding may also be controlled for by including covariates in multivariate analyses. However, it is worth noting that this approach is far less successful than dealing with the covariates (where possible) through experimental means. Including covariates in the analysis reduces the degrees of freedom and thereby the precision of the estimation of experimental error (i.e. power).

### Experimental design and blinding

Several aspects need to be considered when designing an experiment in bioelectromagnetic research. Given the difficulties with heterogeneity as shown in previous chapters, a within-subject design remains the preferred method to reduce the risk of both type I and type II errors. Altogether 33 studies employed a within-subject design (see Table 1). The design involves repeated measurements in the same subject and benefits from the fact that each subject is his/her own control. Yet, participation in one condition may affect performance in other conditions, thus creating a confounding variable that varies with the independent variable. Therefore, subjects should be randomized to the experimental conditions. In the remaining experiments, participants were split into two or three different subgroups (see Table 2). In a between-subject design, the method of randomization creates on average two or more groups that - in theory - are similar with respect to known and unknown factors; the larger the size of the randomly constituted groups, the more likely the groups will be similar. A lot of the error variance in a between-subject design, however, is due to the fact that, even though subjects were randomly assigned to a group they may differ with respect to important individual differences that could affect the dependent or outcome variable. Proper matching reduces this risk and if possible groups should be matched at the subject (individual or one-to-one matching) or at a group level (frequency matching). Moreover, it is advisable to use questionnaires and other methods to collect as much additional information as possible. This increases the chances to identify important differences and to account for them in a statistical model.

It is widely accepted that double-blind experiments are necessary to prevent a possible systematic bias in scientific research. Despite that knowledge a large amount of the reviewed studies were performed single-blindly (see Table 1 and 2). In fact the majority of "positive" findings (i.e., decreased reaction times and/or increased accuracy of performance) resulted from studies employing a single-blind design (see Table 1 and 2) and some results could not be replicated in a double-blind design (e.g. [23,24]). In addition, in a number of studies cognitive performance was assessed while RF EMF exposure and electrophysiological measurements were applied simultaneously (see Table 1). Unfortunately, proper shielding of the recordings system and the amplifier or other precautions applied to ensure that no pick-up of the RF EMF occurred were hardly ever stated. Interference between RF EMF and the recording equipment may lead to a de-blinding of the investigator [18,27,50,58]. One simply cannot overemphasize the importance of applying double-blind conditions; it is absolutely essential in order to gain unbiased data and should be stringently applied in future studies.

## Exposure conditions

### Setup and positioning of the exposure source

Differences in the exposure setup might contribute to inconsistent findings. For example, trying to validate the results of decreased reaction times in the 3-back task reported by Koivisto et al. [14], Haarala et al. [24] used both, a higher average SAR_10 g _and peak SAR in their replication study. Moreover, the occurrence of the peak SAR was closer to the cortex. Besides essentially not being a "real" replication study in the first place, previous results could not be corroborated.

Whereas some experiments included a highly controlled exposure setup (e.g. [11,12]), a large proportion of studies used modified commercial or generic mobile telephones to expose subjects to RF EMF (see Table 1 and 2). Relevant variations exist with respect to telephone model and especially with respect to the positioning of the mobile phone. Several authors simply indicated that it was oriented in normal position of use [17,22,25,28,52], or arranged in direct contact/fixed to the ear [16,27,30,49,59]. Other research groups provided supplementary, but nevertheless highly diverse information regarding the position and/or the distance of the antenna to the head. In some studies, solely antennas were applied for RF EMF exposure [11,12,43]. The huge variability within and between exposure conditions and studies is even higher in experiments in which the phone is not fixed in a predefined position, but has to be held with one hand to one ear by the study participants during exposure (e.g. [29,34,47,57]). Recently, Boutry et al. [62] pointed out that a phone-based setup exposure of the cortex may vary greatly as a function of the setup, position, and local anatomy. This has considerable implications with respect to, e.g. the side of exposure. The antenna of a mobile phone may overlie slightly different brain areas depending on the exposure side (left vs. right side of the head) as the antennas are asymmetric positioned within the phone. Accordingly, slightly different brain areas are maximally exposed in the left and right hemisphere which may therefore result in different effects.

RF EMF exposure was either applied to the left hemisphere, to the right hemisphere or to both hemispheres (see Table 1 and 2). Croft et al. [21] and Fritzer et al. [43] exposed over the posterior cortex and the top of the subjects' head. Though experiments on electrical brain activity suggest that lateralization effects are unlikely (e.g. [2,9]), differences in exposure side may still yield a potential impact with respect to the variability found on cognitive performance. Boutry et al. [62] point to the need for a carefully designed exposure setup that exposes the relevant brain areas to a well-defined level. No standardized exposure setup or procedure exists so far. Thus, in order to minimize the variability within and between subjects, it is in the responsibility of each research group to assure highly reproducible exposure conditions in an experimental environment. Importantly, sufficiently detailed dosimetric information must be provided in order to be able to properly compare different studies and to be able to perform replication or follow-up studies.

### Field conditions and dosimetry

Potential reasons for inconsistencies in bioelectromagnetic research include poorly defined exposure setups, insufficient description of field conditions and the lack of detailed dosimetric data. Very often modified or generic mobile telephones are used to expose subjects to RF EMF. In 16 studies, the brand of the phone was explicitly stated (see Table 1 and 2). Each phone has its unique footprint and thus different types of cell phones may result in different exposure conditions [62]. Though all publications at least briefly name their field conditions, great variability exists with respect to the elaborateness of the description. Differences between studies already emerge when comparing carrier frequencies and the pulse modulation of the signals. The majority of research groups applied GSM signals with a carrier frequency around 900 MHz pulse modulated at 217 Hz or at 2, 8, 217 and 1736 Hz and higher harmonics (see Table 1 and 2). Moreover, a carrier frequency of 450 MHz pulse modulated at 7 Hz [19,33], of 800 MHz pulse modulated at 50 Hz [57], of 1870 MHz pulse modulated at 217 Hz [58], of 1800 MHz [34] and of 1900 MHz [28] RF EMF exposure were studied. UMTS exposure with a carrier frequency of 1950 MHz [63] and 1790 MHz pulse-modulated at 5 MHz [59,61] was investigated in three recent experiments. Some papers only mention the carrier frequency [29,53] or the mean output power [31] in addition to the brand of the mobile telephone [28,34]. Others provide further information regarding the pulse modulation in combination with mean or peak power, pulse width or duty cycle (see Table 1 and 2). Taken together, no publication provides full detail on the field parameters. Yet, some research groups refer to secondary literature for further details (e.g. [11,12,43,63]).

Typically, sham exposure in which no RF EMF is applied resembles the control condition of choice. Besides the need for a control condition without field, the number of exposure conditions should be considered carefully prior to an experiment. The majority of research groups compared a single exposure to sham control (see Table 1 and 2). It may well be possible that additional exposure conditions add important information to the question investigated. Thus, several studies applied two or more exposure conditions and obtained details regarding the effects of modulated or unmodulated signal patterns [11,16,48,50,53,64], varying power levels [52,59], possible dose-response relationships [12] or GSM vs. UMTS exposure [63].

Field conditions are commonly described in various manners. Information on power (W), power density (W/m^2^) or SAR (W/kg) is very difficult to compare, if possible at all. The SAR is an important measure as it specifies the amount of energy absorbed by the underlying biological tissue per units of time and mass. In principle, one can request the maximal SAR for a particular mobile phone from the manufacturer (e.g. [54]) or use corresponding online sources (e.g. [17]; http://www.sarvalues.com/). In general, the SAR values vary between ~0.1-1.9 W/kg. These SAR values apply in case the mobile phone is in direct contact with the head. It was shown that the peak spatial SAR values can vary by more than a factor of 20 from phone to phone within the cortex [62,65]. Moreover, specifying the peak SAR does not provide any information where within the brain it occurs or the shape of the "footprint" of a phone [62]. Indeed a detailed dosimetry (e.g. [65]) is necessary prior to the beginning of an experiment in order to find out how much brain regions of interest are effectively exposed and to draw meaningful conclusions from the results.

### Exposure duration and carryover effects

To date, durations of short term exposure vary from 10-20 minutes [21,33,34] to 150 minutes [35]. Within a sleep study (variable exposure duration of 6.45-8.56 hours during sleep), Fritzer et al. [43] measured cognitive performance before or after a nighttime sleep episode. Besset et al. [47] assessed cognitive performance before, during and after long-term RF EMF exposure (2 hours per day, 5 days per week, 4 weeks). In the majority of experiments cognitive tasks and exposure were carried out simultaneously (see Table 1 and 2) and the duration of the exposure mainly matched the duration of the cognitive tasks employed. As the underlying biological mechanism of RF EMF exposure has not been identified yet, it is difficult to define meaningful exposure durations for an experiment. It may be assumed that very brief exposure periods of only a couple of minutes may be possibly too short to induce an observable effect. This is supported by a recent study indicating that a certain exposure duration might be needed to measure cognitive performance changes [11]. Still, effects have been reported during 10-20 minutes [33,34], 25-30 minutes [11,12,14,16,18,28,29], 40-60 minutes [15,17,19,26,27,31] or 120 minutes of exposure [30] indicating that the chance of detecting an effect does not necessarily seem to increase with increasing exposure duration.

In a crossover design, the time interval between the exposure conditions plays an important role with respect to possible carryover effects, as these may bias a direct exposure effect. If an effect induced by the first exposure condition persists into the second exposure period, it might be attributed to the wrong condition and therefore lead to erroneous results. Several research groups performed two or three consecutive exposures in the same experimental session (see Table 1). Whereas conditions were administered randomly and pseudorandomly in the studies of Schmid et al. [59] and Unterlechner et al. [61], respectively, most research groups applied the conditions in a counterbalanced order (see Table 1 and 2). Yet, nothing is known about a possible effective period of RF EMF exposure. As it might well be that the effects of RF EMF outlast the exposure period (e.g. [11]), the incorporation of a washout period in the design should be always considered to minimize the impact of possible carryover effects. As a precaution, different conditions should be separated by at least 24 hours or longer. Moreover, all test sessions of a subject should be administered with exactly the same time period between sessions and at the same time of day.

## Conclusions

Two recent meta-analyses indicate that short-term RF EMF exposure at handset-intensities may [36] or may not [37] induce subtle cognitive changes. In line with these two publications [36,37] we conclude that several factors must be taken into account which may strongly influence the variance within and between studies focusing on behavioral effects (Table 3). With respect to methodological issues, differences regarding sample size and sample characteristics (e.g. age, gender, special subgroups of participants, inclusion and exclusion criteria), the experimental design (e.g. blinding, carryover effects), the exposure characteristics (intensity, carrier frequency, waveform, exposure duration, side of exposure, distance to source) and dosimetric data (e.g. computer simulation, phantom measurements) have to be considered. Also, the time of testing (during vs. prior and following exposure) as well as the implementation of the number and types of cognitive tasks should be carefully evaluated prior to the beginning of an experiment. Possible confounders (e.g. caffeine and alcohol consumption, altered sleep prior to the experiment) must be controlled for and compliance to predefined requirements (e.g. maintaining a habitual sleep-wake schedule, no caffeine during the study) should be verified (e.g. wrist-worn activity monitors, saliva samples). Double-blind conditions should be applied. In case of simultaneous recording of electrical brain activity and cognitive performance appropriate EMF shielding of the equipment is required unless it is proven otherwise that no pick-up of RF EMF occurred. The study sample should be large enough to reach a power of about 0.80. Minimal requirements with respect to the exposure setup and the exposure conditions involve a sufficient documentation of the setup and technical specifications including the signal (e.g. strength, frequency components) to allow follow-up on an experiment. Also, appropriate dosimetric data with respect to the exposed brain areas has to be provided. For example, 'talk', 'listen' and 'standby mode' signals of GSM mobile phones differ in their ELF spectral composition and SAR values [66,67]. Accordingly, these conditions should be sufficiently specified when applied. A sham control condition, in which no field is applied, should be included for comparison. The application of more than one exposure condition may add important information to the general outcome and even strengthen a result. Moreover, participants should be exposed and tested at the same time of day, but not on the same day, at fixed time intervals to rule out possible time of day and carryover effects.

So far, no specific task has been identified which seems to react especially sensitive to RF EMF exposure. Accordingly, a variety of different tasks are generally implemented in today's exposure studies. A future aim should focus on the identification of sensitive tasks, or types of tasks, in order to gain more insights into possible behavioral consequences of RF EMF exposure. Meanwhile, studies should follow-up on tasks which previously responded to RF EMF exposure and they should be applied under highly controlled conditions. Preferably, more than one task should be included in studies and the tasks should hold a varying degree of difficulty as a task might be simply too easy or too difficult and therefore mask a potential RF EMF induced performance change. Depending on the experimental design, at least one practice session should be scheduled at a fixed time interval (e.g. one week prior to the first experimental session) prior to the experiment to reduce learning effects. In a within-subject design, parallel test forms should be used, or, if not available, the sequence of stimuli should be chosen at random. In general, tasks should be well described to allow replication or follow-up studies and the required responses must be described (e.g. pressing a button on a keyboard with a certain finger). To prevent that the task order per se might mask a potential effect, it is advisable to apply the cognitive tasks in exactly the same order to assure the same amount of elapsed time for each task in the course of exposure.

A valid question remains what impact slight changes in reaction times or accuracy may actually have on real life situations. In fact, it turns out to be very difficult to draw conclusions about abstract mental activity interferences purely on the basis of behavioral data. It may be questioned whether cognitive tests constitute a useful and valid instrument at all in the assessment of RF EMF exposure effects on the brain. Yet, it may be hypothesized that consequences on higher cognitive functions may be even stronger in case elementary motor reactions are influenced.

We strongly support the view of Valentini and colleagues [37] that fully blinded, methodologically detailed and statistically powerful studies are needed and that the involvement of the World Health Organization (WHO) in the development of research standards and guidelines of bio-electromagnetic research would be useful. It is important to stress that nearly all studies up to now assessed changes in cognitive performance after short-term RF EMF exposure. The continuously increasing number of mobile phone users strengthens the need to investigate especially long-term effects of RF EMF of mobile communication systems on brain functioning. Moreover, it would be necessary to include children in future studies. In humans, the studies performed to date mainly assessed the responses to RF EMF exposure in young and middle-aged male and female subjects. Children might represent a specifically sensitive subgroup as their brain is not yet completely mature. Therefore, it is possible that children react differently to RF EMF exposure. However, two recent studies in 10-14 year-old boys [52] could not corroborate influences on cognitive performance reported in adults [14-16]. In addition, it has recently been suggested that special groups of "responders" and "non-responders" to RF EMF exist [21]. Future research may hopefully yield new insights into such interindividual differences within a population. However, in general a more standardized research approach is needed to reveal meaningful results. Only then the relevance of the reported effects can be judged and a risk assessment can be established. The use of worst-case scenarios including appropriate SAR levels may maximize the likelihood to find an effect. At present, no underlying biological mechanism has been identified with respect to the mediated effects observed in the EEG. A future aim will be to identify such mechanisms if existent and to establish a reliable exposure protocol in order to gain more insights into possible behavioral and related health consequences of RF EMF exposure.

## List of abbreviations

CNS: central nervous system; EEG: electroencephalogram; ELF: extremely low frequency; ERP: event related potentials; GSM: Global System for Mobile Communication; non-REM: non-rapid-eye-movement; rCBF: regional cerebral blood flow; RF: radio frequency; RF EMF: radio frequency electromagnetic field(s); SAR: specific absorption rate; UMTS: Universal Mobile Telecommunication System; WHO: World Health Organization.

## Competing interests

This was not an industry supported review. Peter Achermann received previously support from the Swiss Research Foundation on Mobile Communication (FSM), a non-profit foundation sponsored by ETH Zurich, Nokia Siemens Networks, Mobilezone, Orange, Sunrise and Swisscom. He is vice-president of the Foundation for Research on Information Technologies in Society, a non-profit foundation which has on its board personalities from science, the public sector and the global wireless communications industry.

## Authors' contributions

PA obtained the funding for this review. SJR drafted the manuscript and PA substantially contributed to the draft and reviewed it critically. Both authors revised and approved the final version of the manuscript.
